# Identification of a Novel *Alternaria alternata* Strain Able to Hyperparasitize *Puccinia striiformis* f. sp. *tritici*, the Causal Agent of Wheat Stripe Rust

**DOI:** 10.3389/fmicb.2017.00071

**Published:** 2017-01-31

**Authors:** Li Zheng, Jie Zhao, Xiaofei Liang, Gangming Zhan, Shuchang Jiang, Zhensheng Kang

**Affiliations:** State Key Laboratory of Crop Stress Biology for Arid Areas and College of Plant Protection, Northwest A&F UniversityYangling, China

**Keywords:** wheat stripe rust, *Puccinia striiformis*, hyperparasite, *Alternaria alternata*, biological control

## Abstract

The obligate bitrophic fungus *Puccinia striiformis* f. sp. *tritici* (*Pst*) causes stripe (yellow) rust on wheat worldwide. Here, we report a novel fungal strain able to hyperparasitize *Pst*. The strain was isolated from gray-colored rust pustules, and was identified as *Alternaria alternata* (Fr.: Fr.) keissler based on a combination of morphological characteristics and multi-locus (ITS, GAPDH, and RPB2) phylogeny. Upon artificial inoculation, the hyperparasite reduced the production and viability of urediniospores, and produced a typical gray-colored rust pustule symptom. Scanning electron microscopy demonstrated that the strain could efficiently penetrate and colonize *Pst* urediniospores. This study first demonstrates that *A. alternata* could parasitize *Pst* and indicates its potential application in the biological control of wheat stripe rust disease.

## Introduction

Strip rust (yellow rust), caused by *Puccinia striiformis* Westend f. sp. *tritici* Erikss. (*Pst*), is one of the most important diseases of wheat in many regions of world (Saari and Prescott, 1985; Stubbs, 1985; Chen, 2005). Because the *Pst* urediniospores could be dispersed over long distances by the wind, the fungal pathogen is able to cause large-scale epidemics and severe yield losses under conducive environmental conditions (Brown and Hovmøller, 2002; Wan et al., 2007; Zhao et al., 2008). In China, the devastating epidemics occurring in 1950, 1964, 1990, and 2002, has caused up to 6.0 × 10^9^, 3.0 × 10^9^, 2.6 × 10^9^, and 1.0 × 10^9^ kg of yield losses, respectively (Wan et al., 2004; Chen et al., 2009; Gao et al., 2015). So far, cultivation of resistant varieties is the most effective way to control wheat stripe rust. Nevertheless, most resistant varieties were bred for major gene resistance and rapidly lost their resistance within 3–6 years after field cultivation (Cheng et al., 2014; Han et al., 2015). In addition, the constant and indiscriminate use of fungicides poses serious environmental problems and health hazards to animals and humans. Biological control strategy is thus attractive for the potential to achieve effective disease management with minimal environmental cost.

*Puccinia striiformis* f. sp. *tritici* (*Pst*) is an obligate biotrophic fungus, which normally forms yellow to orange urediniospores on leaf blade surfaces during disease progression (Hovmøller et al., 2011). However, we have observed that the color appearance of uredinia (urediniospore mass) occasionally turn dark gray overtime during greenhouse propagation, especially under high humidity conditions. The color shift takes place gradually and becomes increasingly common in frequency, which finally causes the cessation of uredinia sporulation. Our previous study has demonstrated that such discoloration and sporulation cessation could be associated with hyperparasite infection (Zhan et al., 2014).

Hyperparasitism is common in filamentous fungi, and could be developed into a useful alternative to chemical fungicides for effective control of plant fungal diseases (Hijwegen and Buchenauer, 1984; Blakeman, 1992; McLaren et al., 1996; De Cal et al., 2008; Adhikari et al., 2014; Zhong et al., 2016). Previous studies showed that approximately 30 genera of fungi can hyperparasitize rust pathogens, which included *Tuberculina* spp. (Mijušković and Vučinić, 2001), *Darluca filum* (Yuan et al., 1999), *Fusarium* spp. (Kapooria and Sinha, 1969), *Scytalidium uredinicola* (Tsuneda et al., 2011), *Aphanocladium album* (Koç and Défago, 2008) and *Cladosporium* spp. (Moricca et al., 2001). However, so far only four species, *Cladosporium cladosporioides*, *Lecanicillium lecanii*, *Microdochium nivale*, and *Typhula idahoensis*, have been reported to infect uredinia and urediniospores of *Pst* (Littlefield, 1981; Zhan et al., 2014).

Here we describe the discovery of a novel *Pst* hyperparasite. Morphological observations and phylogenetic analysis demonstrated that the fungus belongs to the species of *Alternaria alternata* (Fr.: Fr.) keissler, which has never been reported to parasitize *Pst* or any other fungal organisms. Pathogenicity test and microscopic examination showed that the obtained *A. alternata* strain is able to reduce *Pst* urediniospore production and viability, which indicates a biological control potential of this novel mycoparasite against wheat stripe rust disease.

## Materials and Methods

### Isolation and Purification of the Mycoparasite

The hyperparasitic strain CPA001 was isolated from *Pst* urediniospores in Northwest A&F University, Yangling, Shaanxi, China. Firstly, urediniospores of *Pst* were propagated on the susceptible wheat cultivar Mingxian 169 as previously described (Cao et al., 2008). Wheat seedlings inoculated with urediniospores of *Pst* were kept in a growth chamber at about 16°C and 80–90% relative humidity (Lu et al., 2011). In total, 151 seedling plants were inoculated. Fourteen to twenty days after inoculation, more than half of the uredinia changed color from fresh yellow orange to gray or dark gray. Gray urediniospores were then transferred onto potato dextrose agar (PDA) medium with a sterilized needle. After incubation at 25° for 3 days, mycelia from the colony margins were transferred to fresh PDA plate and singe-spore purified to obtain a pure culture. The purified culture was stored on PDA slants at 4–8°C.

### Morphological Observation

For the morphological observation, mycelial disks of 5 mm in diameter were taken from the growing margins of 3-day old PDA culture, transferred to potato carrot agar (PCA) plates and incubated at 25°C in a 12-h photoperiod for 1 week to induce conidia production. In addition, microscope slide cultures were prepared by placing a small amount of mycelia on PDA medium blocks (5 mm diameter) overlaid by a cover slip (Wang et al., 2015). Examination of the morphological characteristics of hyphae, conidiophores and conidia were conducted using an Olympus BX51T-32P01 optical microscope.

To further observe the ultrastructure of the parasitic fungus, wheat leaves bearing uredinia with abnormal colors were cut into pieces approximately 0.5 cm × 0.5 cm in size for scanning electronic microscope (SEM). Samples were immersed in 4.0% glutaraldehyde (pH 6.8) and fixed at 4°C for 4 h. Then samples were washed four times with 0.1 M phosphate buffer for 15 min each. Subsequently, samples were dehydrated for 30 min each in 30, 50, 70, 80, and 90% ethanol series, and finally 3 repeats in 100% ethanol. Samples were dried in a CO_2_ vacuum, and sputter coated with gold (E-1045, Hitachi, Japan) for SEM examination (S-4800, Hitachi, Japan).

### Pathogenicity Test to Confirm Hyperparasitism

The susceptible wheat cultivar Mingxian 169 was used for propagating *Pst* urediniospores. When the first leaf had successfully expanded after 10 days, seedlings were inoculated with CYR32, a predominant race of *Pst* in China. Approximately 14 days later, the diseased leaves bearing urediniospores were inoculated with the mycoparasite *A. alternata* strain CPA001. The pure culture of CPA001 was formulated into spore suspension (1.0 × 10^6^ spores/mL in concentration) for spray inoculation. Healthy wheat leaves receiving *A. alternata* inoculation represented control check1 (CK1). Wheat leaves infected by *Pst* but not treated with the *A. alternata* conidia suspension represented CK2. Each treatment was carried out with wheat seedlings growing in three independent pots, with each pot containing about 24 plants. All treatments were placed in the same growth chamber, and observation of the symptoms was performed at the same time.

Simultaneously, *Pst* urediniaspores were directly inoculated with the *A. alternata* strain CPA001. The *A. alternata* conidia suspension (1.0 × 10^6^ spores/mL in concentration) was mixed with the *Pst* urediniospores, the spore mixture was sprayed on PDA medium, and incubated at 25° for 24 h. During co-cultivation, samples were collected to observe the dynamic infection process with SEM. Ultrastructural sample treatments were the same as ones described above.

### Uredinia Quantification

The phenotype of disease was quantitively assessed by counting the number of uredinia pustules within a 5 cm^2^ area at 9 days post CPA001 inoculation, using I_MAGE_J^1^. To avoid bias among leaf samples, 35 random leaves were selected for each treatment and the entire experiment was repeated for three times.

### Germination Rate of *Pst*

Freshly collected urediniospores were cultured on sterile water at 9°C for 6 h, then placed on slides to count the numbers of germinated urediniospores using an Olympus BX51T-32P01 optical microscope. A germ tube length up to the one-half spore diameter was defined as germination. The germination rate was expressed as a percentage based on 100 urediniospores. One hundred urediniospores were selected randomly, and all experiments were performed at least three times.

### Molecular Characterization

#### DNA Extraction

The strain CPA001 was cultured on cellophane placed on top of PDA medium and incubated at 25°C for 7 days, the mycelia were then harvested for DNA extraction. Genomic DNA was extracted with CTAB method described by Wang et al. (2015). DNA concentration was measured with a spectrophotometry (Nanodrop 2000, Thermo Fisher Scientific, Wilmington, DE, USA). The DNA was stored at -20°C and diluted to 100 ng/μL as the working solution for polymerase chain reaction (PCR) amplification.

#### PCR Amplification and Sequencing

Three representative genes [the complete rDNA-ITS (ITS) region, glyceraldephyde-3-phosphate dehydrogenase (GAPDH) and the second largest subunit of RNA polymerase II (RPB2)] of *A. alternata* were amplified using gene-specific PCR primers of the V9G (5′-TTACGTCCCTGCCCTTTGTA-3′) (Hoog and Gerrits van den Ende, 1998) and ITS4 (5′-TCCTCCGCTTATTGATATGC-3′) (White et al., 1990), gpd1 (5′-CAACGGCTTCGGTCGCATTG-3′) and gpd2 (5′-GCCAAGCAGTTGGTTGTGC-3′) (Berbee et al., 1999) and RPB2-5F2 (5′-GGGGWGAYCAGAAGAAGGC-3′) (Sung et al., 2007) and fRPB2-7cR (5′-CCCATRGCTTGTYYRCCCAT-3′) (Liu et al., 1999), respectively. Conditions for PCR amplification of the three genes were as follows: initial denaturing at 94°C for 5 min; 35 cycles of denaturing (each cycle at 94°C for 30 s), annealing at 55°C for 30 s, and extension at 72°C for 1 min; and then a final extension at 72°C for 10 min. PCR products were detected by 1.0% agarose gel electrophoresis, purified using a PCR Purification Kit (Bio-tek Co., Ltd, China) according to the manufacture’s protocol. The amplified products were cloned into pMD20-T vector (Takara) for Sanger sequencing. All sequences were deposited in GenBank under accession numbers KX976465, KX976466, and KX976467, respectively.

### Phylogenetic Analysis

Reference sequences from other *Alternaria* spp. were retrieved from GenBank (**Table 1**). Sequences were aligned with Clustal X (Thompson et al., 1997), and the final alignment was inspected with BioEdit 5.0.9.1 (Saitou and Nei, 1987). On the basis of the aligned sequences, a phylogenetic tree was constructed with the Maximum Likelihood (ML) method in the Molecular Evolutionary Genetics Analysis (MEGA) software version 6.0 with 1000 bootstrap replicates (Efron et al., 1996; Tamura et al., 2013).

**Table 1:** Sources of *Alternaria* spp. strains with GenBank accession numbers.

Species name	Strain number	Gene and GenBank accession no.			Substrate/Host	Origin/Locality
		ITS^a^	GAPDH^b^	RPB2^c^		
*A. alternantherae*	CBS124392	KC584179	KC584096	KC584374	*Solanum melongena*	China
*A. iridiaustralis*	CBS118404	KP124434	KP124283	KP124904	*Iris* sp.	New Zealand
	CBS118486	KP124435	KP124284	KP124905	*Iris* sp.	Australia
	CBS118487	KP124436	KP124285	KP124906	*Iris* sp.	Australia
*A. betae-kenyensis*	CBS118810	KP124419	KP124270	KP124888	*Beta vulgaris* var. *cicla*	Kenya
*A. eichhorniae*	CBS489.92	KC146356	KP124276	KP124895	*Eichhornia crassipes*	India
*A. burnsii*	CBS107.38	KP124420	JQ646305	KP124889	*Cuminum cyminum*	India
	CBS110.50	KP124421	KP124271	KP124890	*Gossypium* sp.	Mozambique
	CBS118816	KP124423	KP124273	KP124892	*Rhizophora mucronata*	India
*A. tomato*	CBS103.30	KP124445	KP124294	KP124915	*Solanum lycopersicum*	Unknown
	CBS114.35	KP124446	KP124295	KP124916	*Solanum lycopersicum*	Unknown
*A. jacinthicola*	CBS878.95	KP124437	KP124286	KP124907	*Arachis hypogaea*	Mauritius
	CBS133751	KP124438	KP124287	KP124908	*Eichhornia crassipes*	Mali
	CPC25267	KP124439	KP124288	KP124909	*Cucumis melo* var. *indorus*	Unknown
*A. alternata*	CBS102599	KP124330	KP124185	KP124798	*Minneola tangelo*	Turkey
	CBS107.53	KP124305	KP124162	KP124774	*Pyrus pyrifolia*	Japan
	CBS115200	KP124352	KP124206	KP124820	*Minneola tangelo*	South Africa
	CBS115616	AF347031	AY278808	KC584375	*Arachis hypogaea*	India
	CBS117143	KP124355	KP124209	KP124823	*Capsicum annuum*	Italy
	CBS118812	KC584193	KC584112	KC584393	*Daucus carota*	USA
	CBS118814	KP124357	KP124211	KP124825	*Solanum lycopersicum*	USA
	CBS118815	KP124358	KP124212	KP124826	*Solanum lycopersicum*	USA
	CBS121348	KP124367	KP124219	KP124836	*Platycodon grandiflorus*	China
	CBS127671	KP124381	KP124233	KP124851	*Stanleya pinnata*	USA
	CBS127334	KP124380	KP124232	KP124850	soil	USA
	CBS121456	KP124369	KP124221	KP124839	*Sanguisorba officinalis*	China
	CBS126910	KP124379	KP124231	KP124849	*Stanleya pinnata*	USA
	CBS795.72	KP124309	KP124166	KP125085	*Plantago aristida*	USA
	CBS620.83	KP124315	KP124171	KP124783	*Nicotiana tabacum*	USA
*A. alternata*	CPA001^d^	KX976465	KX976466	KX976467	*Puccinia striiformis*	China
	CBS102600	KP124331	KP124186	KP124799	*Citrus reticulata*	USA
	CBS115069	KP124347	KP124201	KP124815	*Malus domestica*	South Africa
	CBS119543	KP124363	KP124215	KP124831	*Citrus paradisi*	USA
*A. gaisen*	CBS632.93	KC584197	KC584116	KC584399	*Pyrus pyrifolia*	Japan
	CBS118488	KP124427	KP124278	KP124897	*Pyrus pyrifolia*	Japan
	CPC25268	KP124428	KP124279	KP123976	Unknown	Portugal
*A. arborescens SC*	CBS101.13	KP124392	KP124244	KP124862	Peat soil	Switzerland
	CBS105.24	KP124393	KP124245	KP124863	*Solanum tuberosum*	Unknown
	CBS116329	KP124405	KP124257	KP124875	*Malus domestica*	Germany
	CBS105.49	KP124396	KP124248	KP124866	Contaminant blood culture	Italy
	CBS126.60	KP124397	KP124249	KP124867	Wood	UK
	CBS109730	KP124399	KP124251	KP124869	*Solanum lycopersicum*	USA
	CBS112749	KP124401	KP124253	KP124871	*Malus domestica*	South Africa
	CBS112633	KP124400	KP124252	KP124870	*Malus domestica*	South Africa
*A. arborescens* SC	CBS117587	KP124406	KP124258	KP124876	*Brassica* sp.	Netherlands
	CBS118389	KP124407	KP124259	KP124877	*Pyrus pyrifolia*	Japan
	CBS123266	KP124411	KP124262	KP124881	Human toenail	Denmark
	CBS127263	KP124417	KP124268	KP124886	Human nasal infection	Mexico
	CBS115516	KP124403	KP124255	KP124873	*Malus domestica*	South Africa

*^a^ITS complete rDNA-ITS region; ^b^GAPDH glyceraldephyde-3-phosphate dehydrogenase; ^c^RPB2 the second largest subunit of RNA polymerase II; ^d^Sequences from isolates of A. alternatea from Puccinia striiformis.*

## Results

### Isolation of an *Alternaria alternata* Strain from *Pst* Uredinia Showing Mycoparasitic Symptom

**FIGURE 1:**
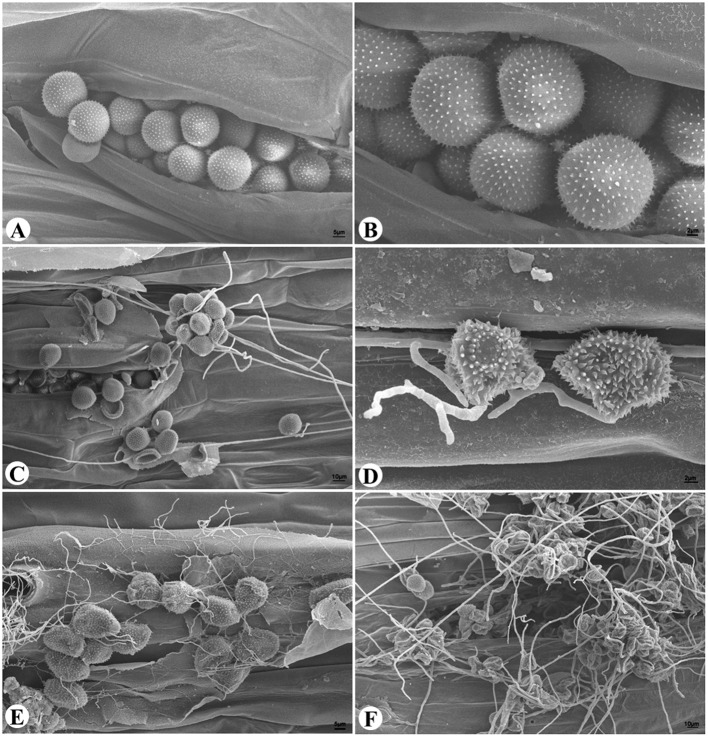
**The hyperparasitic colonization of *Pst* uredinia and urediniospores. (A,B)** Uredinium and urediniospores in normal shape. **(C)** Early stage of hyperparasitic infection, note the shriveled urediniospores. **(D,E)** Mid infection stage. **(F)** Late infection stage.

Wheat leaves bearing normal yellow-colored uredinia and leaves bearing gray-colored uredinia resembling hyperparasite infection were examined under SEM (**Figure 1**). Yellow-colored uredinia contained round-shaped urediniospores, and contained no mycelium or spore of other fungal organism (**Figures 1A,B**). On the other hand, gray-colored uredinia were made up of shriveled or ruptured urediniospores, these spores were intertwined by dense filamentous hyphae, which become increasingly prevalent over time (**Figures 1C–F**). The dramatic shape change of urediniospores indicated their loss of cell viability. The prevalence of intertwined hyphae strongly suggested hyperparasitic colonization events.

**FIGURE 2:**
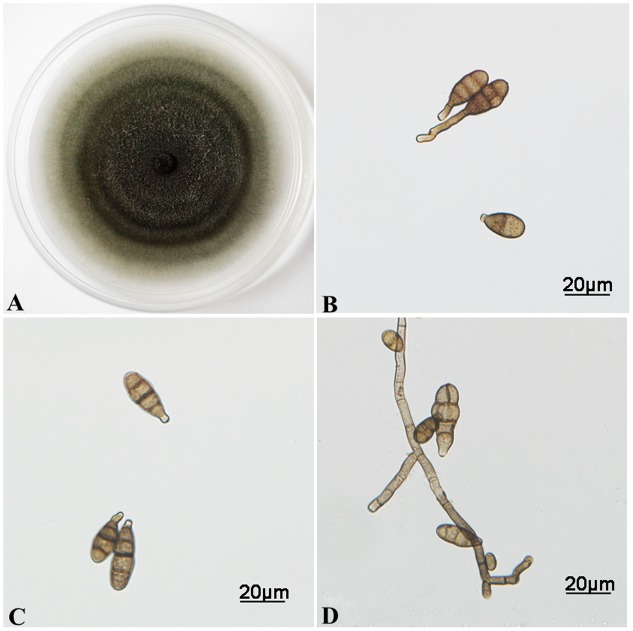
**Morphological characterizations of *A. alternata* cultured on PCA medium. (A)** Colony morphology grown at 25°C for 7 days. **(B–D)** Conidia and vegetative hyphae morphology.

**FIGURE 3:**
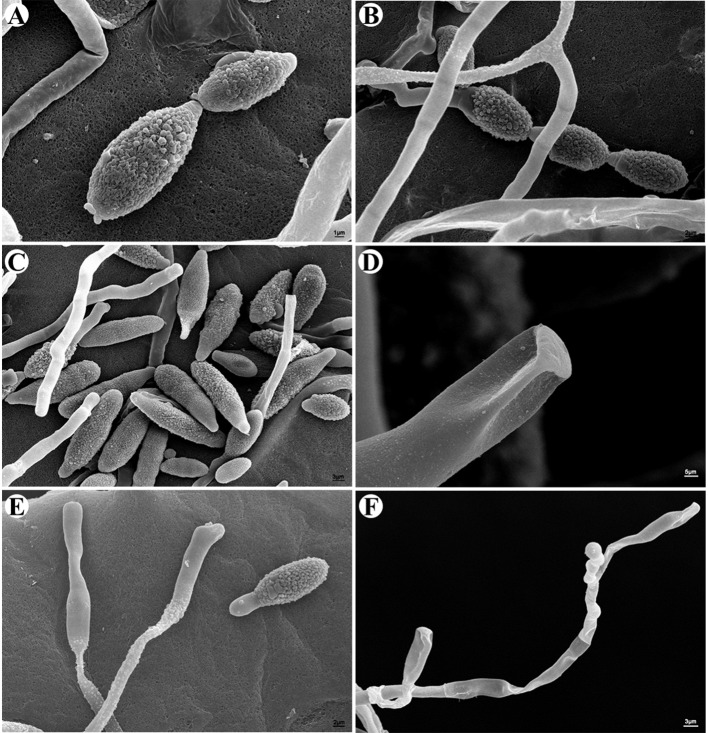
**Morphological characterizations of *A. alternata* under SEM. (A–C)** Conidia. **(D)** Scars on a secondary conidium. **(E,F)** Conidiophores.

Through *in vitro* culture, several candidate mycoparasitic fungal strains were obtained. Most strains resembled the previously reported *C. cladosporioides* in morphological appearance (Zhan et al., 2014), which were not characterized further. One strain, named CPA001, was characterized further and reported here. On PCA medium, the CPA001 culture initially developed light-gray colony and the center turned dark gray after 7 days (**Figure 2A**). The vegetative hyphae were brown, branched, septate, and 4 μm in diameter (**Figure 2D**). Conidia were typically obpyriform, dark brown, 20.2 to 35.2 μm × 8.0 to 12.6 μm in size, with 1–3 transverse and 0–1 longitudinal septate. Most conidia also had a short beak with a dimension of 1.6 to 9.4 μm × 2.9 to 5.0 μm (**Figures 2B–D**). SEM observation obtained more detailed morphological characteristics of the conidia and conidiophores (**Figures 3A–F**). SEM observations indicated that the fungus produced abundant conidia in long chains on short conidiophores (**Figures 3A–C**). Conidiophores were single, straight or slightly curved, and ranged from 12.3 to 60.6 μm × 2.2 to 4.0 μm (**Figures 3E,F**). These morphological features resembled *Alternaria* spp.

**FIGURE 4:**
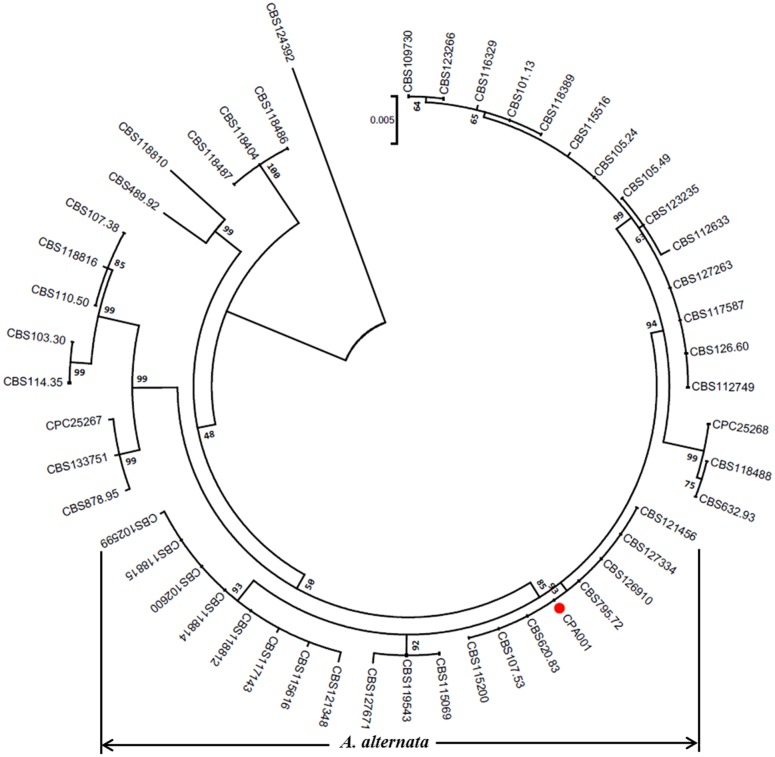
**A phylogenetic tree constructed based on the three genes (ITS, GAPDH and RPB2) of the members in *Alternaria* genus using the maximum likelihood (ML) method with 1000 bootstrap replicates.** The red circle represents the *A. alternata* isolate characterized in the present study.

CPA001 was further identified to be *A. alternata* based on phylogenetic analysis with the ITS, GAPDH and RPB2 genetic markers (**Figure 4**). A range of *Alternaria* spp. reference isolates were selected for the phylogenetic tree construction. CPA001 was found to be most closely related to CBS121456, CBS127334, CBS126910,CBS795.72, CBS620.83, CBS107.53 and CBS115200, all belonging to the *A. alternata* species. The entire *A. alternata* clade was well-separated from other clades with a bootstrap value of 85%.

### Confirmation of Hyperparasitism

**FIGURE 5:**
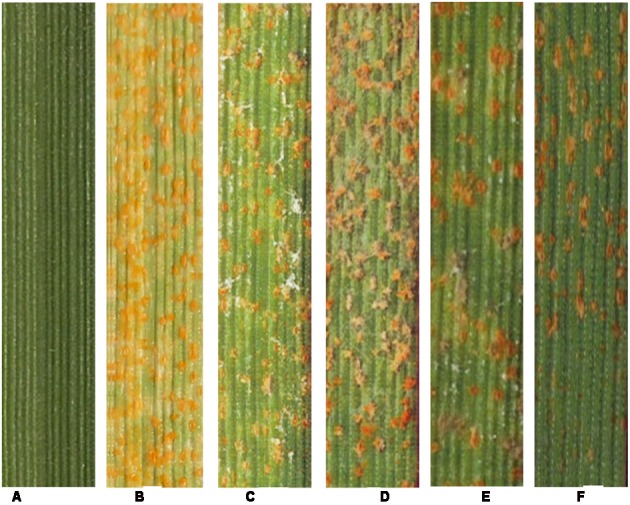
**Pathogenicity test to confirm that *A. alternata* could hyperparasite *Pst*. (A)** CK1, wheat leaves inoculated with the spore suspension of *A. alternata*, 20 dpi, without any symptom; **(B)** CK2, wheat leaves only inoculated with CYR32, 20 dpi; **(C–F)** Wheat leaves inoculated with CYR32 for 14 days prior to inoculating with the spore suspension of *A. alternata*. **(C–F)** are symptoms at 3, 5, 7, and 9 d after *A. alternata* inoculation respectively.

**FIGURE 6:**
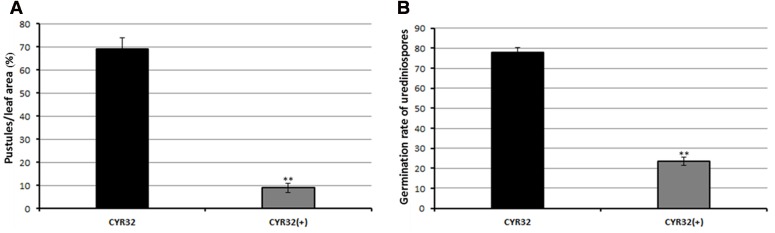
**Quantification of the percentage of leaf area covered by *Pst* pustules (A)** and germination rate of *Pst* urediniospores **(B)**. CYR32 (+) means mycoparasite infected. Values represent mean ± standard errors of three independent assays, and the statistical analysis was assessed by using Student’s *t*-tests. Double asterisks indicate *P* < 0.01.

Pathogenicity testing showed that the obtained CPA001 strain could efficiently hyperparasitize *Pst* (**Figure 5**). Wheat leaves inoculated with *Pst* alone produced abundant orange-colored uredinia after 21 days post inoculation (**Figure 5B**). On the other hand, *Pst* pre-inoculated wheat leaves receiving subsequent *A. alternata* treatment showed a typical sign of mycoparasitic colonization, namely fewer rust pustule formation and abundant gray-colored hyphae covering the uredinia (**Figures 5C–F**). Such gray-colored hyphae were never observed with wheat leaves treated with *A. alternata* conidia suspension alone (CK1, **Figure 5A**). Reisolated strain from the parasitized uredinia showed the same morphological characteristics CPA001. At 9 days post CPA001 inoculation, the frequency of *Pst* pustule formation was merely 10% whereas that of the control treatment was 70% (**Figure 6A**). Urediniospores collected from CPA001-treated pustules also showed dramatically reduced viability (∼25% vs. 80%), indicated by germination rate (**Figure 6B**).

**FIGURE 7:**
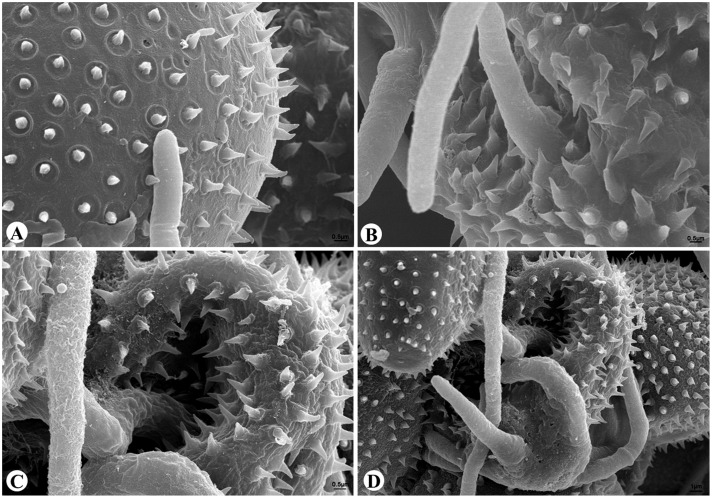
**Scanning electronic microscope observations of *Pst* urediniospore infection by *A. alternata*. (A)** 24 h after inoculation, the *A. alternata* germ tube contacted with *Pst* urediniospore; **(B)** 36 h after inoculation, an *A. alternata* germ tube penetrated into a urediniospore; **(C,D)** 48 h after inoculation, the hyphae of *A. alternata* directly penetrated through the urediniospore.

Scanning electronic microscope observations further confirmed that the *A. alternata* CPA001 strain could efficiently parasitize *Pst*. The *A. alternata* germ tube contacted with and penetrated into *Pst* urediniospores at 24 hpi, and caused complete urediniospore collapse at 36–48 hpi (**Figures 7A–D**).

## Discussion

Characterization of newly isolated mycoparasites has contributed to a better understanding of the diversity of hyperparasites, and will lead to the discoveries of novel fungal species and the development of novel biocontrol agents (Vandermeer et al., 2009; Baiswar et al., 2014; Wang et al., 2015; Zhong et al., 2016). The present study revealed a novel mycoparasite infecting *Pst*, the causal agent of wheat stripe rust. In addition, the hyperparasite could reduce the production and viability of urediniospores, indicating its potential application in the biological control of *Pst.*

Conidial morphology and size used to be important features used in *Alternaria* taxonomy. However, these phenotypes are plastic, showing considerable variations under different environmental and culture conditions, making it difficult to identify species based on phenotype alone (Rotem, 1994). Although ITS region of nuclear ribosomal DNA (nrDNA) is a universal marker used for the identification of fungal species (Seena et al., 2010; Schoch et al., 2012), it is ineffective in distinguishing closely related fungal species (Pryor and Gilbertson, 2002; Kiss, 2012). Currently, multiple gene-based phylogeny has been widely used in the classification of species in the genus *Alternaria* (Andrew and Pryor, 2008; Lawrence et al., 2013; Woudenberg et al., 2015). In the present study, a combination of three markers identified the obtained mycoparasite to be *A. alternata*. To our knowledge, this is the first report that *A. alternata* could hyperparasitize *Pst*, and also the first report of *A. alternata* as a hyperparasite.

Resistance breeding is critical for wheat rust disease control. Nevertheless, most resistant genes used for breeding are ones with major effect, which tend to lose their resistance rapidly upon field release (Line and Qayoum, 1992; Li and Zeng, 2000). So far, mycoparasitism has been reported as an effective measure for controlling several diseases (Zhong et al., 2016). For example, *Trichoderma* spp. has been successfully used to minimize the effect of *Fusarium oxysporum* pathogen on tomato plants (Adhikari et al., 2014). *Ampelomyces quisqualis* is in commercial use for biocontrol of powdery mildew on grapes and other crops (Sullivan and Maddock, 2000). However, there have been little known attempts to control *Pst* with hyperparasites.

The *A. alternata* strain CPA001 obtained in the present study can colonize *Pst* urediniospores in an aggressive manner. CPA001 treatment dramatically reduces uredinial pustule formation and the viability of ureniniospores. Moreover, our observation indicated that CPA001 can colonize a broad range of *Pst* isolates being different in virulence profile (physiological race) and geographic origin. These facts make CPA001 a good candidate for further characterization efforts to develop novel *Pst* biocontrol agent. But now, we are unclear about the mycoparasitism spectrum of CPA001 at a broader level (e.g., its hypoparasitic potential against other rust pathogens), and whether and how environmental factors affect the survival ability and hypoparasitic potential of CPA001. We also do not know by which strategies CPA001 kills and colonizes *Pst* urediniospores and whether these strategies are CPA001-unique or are general features of the *A. alternata* species. In the near future, it is important to study the hypoparasitic characteristics of CPA001 in more detail in the laboratory, so as to understand its parasitism spectrum, its hypoparasitic mechanisms, and the potential environmental and ecological impacts upon massive release. Key factors impacting mycoparasitism efficiency should be identified and controlled field test should be performed to determine the disease control effect.

Urediniospores are important inoculation materials for rust disease research. Based on our experience, mycoparasitic infection of *Pst* is common in the greenhouse, which could pose a great challenge to research activities such as spore propagation. In the near future, we are planning to further characterize the biological characteristics of the obtained mycoparasitic isolate, such as the experimental host range, the spore type specificity, and the effects of environmental conditions (e.g., humidity, moisture) on the final parasitic infection outcome. These efforts will offer important principle guidelines for the field application and greenhouse control of *Pst* mycoparasites.

## Author Contributions

ZK designed experiments; LZ performed the experiments; JZ and XL analyzed the data; GZ and SJ joined the discussion and gave the original ideas; LZ wrote the paper.

## Conflict of Interest Statement

The authors declare that the research was conducted in the absence of any commercial or financial relationships that could be construed as a potential conflict of interest.

## Footnotes

1 http://rsb.info.nih.gov/ij
